# Investigating the Impact of *ABCB1 3435C>T* (rs1045642) Variant on Severity and Cognitive Decline in Egyptian Alzheimer’s Disease Patients

**DOI:** 10.1007/s12035-026-05789-w

**Published:** 2026-03-26

**Authors:** Yasmeen K. Farouk, Nahla Elsayed Nagy, Azza M. El Amir, Neveen Adel Madbouly, Nashwa El-Khazragy

**Affiliations:** 1 Egypt Center for Research and Regenerative Medicine (ECRRM), Cairo, 11599 Egypt; 2 Department of Neuropsychiatry, Faculty of Medicine, Ain Shams University, Cairo, 11566 Egypt; 3 Department of Biotechnology, Faculty of Science, Cairo University, Giza, 12613 Egypt; 4 Department of Zoology, Faculty of Science, Cairo University, Giza, 12613 Egypt; 5 Department of Clinical Pathology-Hematology and AinShams Medical Research Institute (MASRI), Faculty of Medicine, Ain Shams University, Cairo, 11566 Egypt

**Keywords:** Alzheimer’s disease, *ABCB1 3435C>T* (rs1045642), Cognitive impairment, Dementia

## Abstract

Alzheimer’s disease (AD) is a progressive neurodegenerative disorder with a complex genetic risk. The *ABCB1* gene encodes *P-glycoprotein*, a blood-brain barrier efflux transporter involved in *amyloid-β* (*Aβ*) clearance. Variants in *ABCB1 3435C>T* (rs1045642) may influence AD susceptibility and severity, but data from Middle Eastern populations remain limited. The objective of the study is to investigate the *ABCB1 3435C>T* (rs1045642) polymorphism with AD susceptibility, dementia severity, and cognitive impairment in an Egyptian cohort. A case–control study was conducted on 300 subjects, including 150 cognitively healthy controls and 150 clinically diagnosed AD patients. Genotyping for *ABCB1 3435C>T* (rs1045642) was performed by polymerase chain reaction (PCR). Associations between genotype distribution and AD risk, Clinical Dementia Rating (CDR) scores, and Mini-Mental State Examination (MMSE) categories were analyzed. Logistic regression and Fisher’s exact tests were applied to calculate odds ratios (OR) and 95% confidence intervals (CI). The CC genotype was significantly more frequent among controls (64.8%) compared with AD patients (35.2%), whereas the TT genotype predominated in AD (70.5% vs 29.5%). CC carriers were enriched in mild dementia and higher MMSE categories, while TT carriers dominated in severe dementia and lower MMSE groups. Logistic regression confirmed that TT carriers had a 4.4-fold increased risk of AD compared with CC (*p* < 0.001) and a 13.3-fold higher likelihood of scoring < 20 on MMSE (*p* < 0.001). The *ABCB1 3435C>T* (rs1045642) variant significantly influences AD susceptibility and cognitive decline in Egyptians, with TT conferring risk and CC exerting a protective effect. Genotyping may aid risk stratification and monitoring of disease progression.

**Graphical Abstract:**

Association of *ABCB1 3435C>T* (rs1045642) variant with AD severity and neuropsychological symptoms. The *ABCB1 3435C>T* (rs1045642) variant was associated with increased AD severity and more pronounced neuropsychological symptoms among Egyptian patients, suggesting a potential genetic influence on disease progression and symptom manifestation

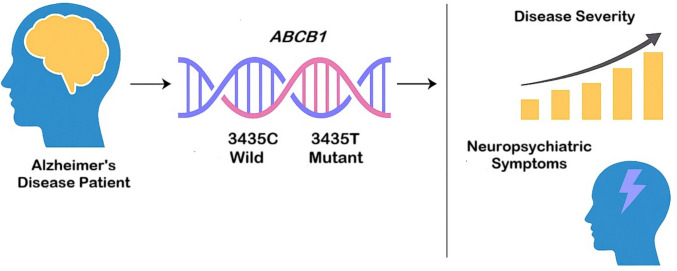

## Introduction

Alzheimer’s disease (AD) stands as the predominant cause of dementia globally, representing a mounting public health concern that intensifies with the progressive aging of the population [1, 2]. Currently, over 55 million individuals are affected by dementia worldwide, and estimates suggest that this number may almost triple by 2050, with AD responsible for most of these cases [3, 4]. In Egypt, epidemiological surveys estimate dementia prevalence among elderly populations at around 4–5% [5], with AD constituting the dominant type [6–8]. Beyond cognitive decline [9], AD manifests through a broad range of neuropsychological symptoms [10], such as depression, anxiety [11], agitation, psychosis [12], and sleep disturbances that significantly affect patients’ quality of life, increase caregiver burden, and pose challenges to community healthcare systems [13]. These symptoms not only diminish patients’ quality of life but also heighten caregiver stress and create substantial challenges for community healthcare services [14], earlier institutionalization, and heightened morbidity, thus underscoring their importance in disease assessment and management [14, 15].

Despite decades of intensive investigation, Alzheimer’s disease (AD) continues to be an irreversible and progressively worsening condition [16]. Existing treatments such as cholinesterase inhibitors and N-methyl-D-aspartate (NMDA) receptor antagonists [17] offer limited symptomatic improvement but fail to stop or reverse the underlying neurodegenerative process [18, 19]. Multiple interconnected pathological processes contribute to the development of AD [20–22], including the accumulation of *Aβ* plaques [23], abnormal hyperphosphorylation of tau proteins [24], elevated oxidative stress [23], impairments in mitochondrial function [25, 26], neuroinflammation, and vascular alterations [27], which drive disease progression [28]. As such, emerging approaches aim to delay decline by targeting modifiable risk factors, optimizing pharmacological and non-pharmacological interventions, and exploring personalized medicine strategies [29, 30]. A deeper understanding of genetic determinants of AD risk and progression offers promise in identifying vulnerable populations and guiding targeted interventions [31–33].

Genetic susceptibility is a well-established contributor to AD pathogenesis [34, 35]. Mutations in the *APP*, *PSEN2*, and *PSEN1* genes are strongly associated with familial early-onset AD [36, 37], while the more prevalent sporadic late-onset form arises from a combination of genetic and environmental influences, most notably the presence of the apolipoprotein E (*APOE*) ε4 allele [38, 39]. Beyond these canonical genes, several other loci and single-nucleotide polymorphisms (SNPs) are increasingly recognized for their roles in modulating AD susceptibility, progression, and clinical heterogeneity [40, 41]. Variants that regulate neuroinflammatory pathways [42], synaptic function, and drug transport systems are of particular interest, as they may shape both disease expression and treatment outcomes [43].

The *ABCB1* gene, commonly referred to as the multidrug resistance gene 1 (*MDR1*), codes for *P-glycoprotein* (*P-gp*) [44], a transmembrane efflux transporter primarily situated at the blood-brain barrier (BBB) [45]. Situated on chromosome 7q21.12 [46], *ABCB1* has a major role in regulating the entry and clearance of xenobiotics, endogenous metabolites, and therapeutic agents from the brain [47]. *P-gp* influences *Aβ* efflux across the BBB, thereby linking it directly to AD pathophysiology [48]. Dysfunction or reduced expression of *P-gp* may contribute to amyloid accumulation, exacerbating neurodegeneration [49, 50]. Moreover, *ABCB1* activity intersects with key signaling pathways, including xenobiotic metabolism, oxidative stress regulation, and neuroinflammatory cascades, all of which are implicated in neurodegenerative disease mechanisms [51].

Numerous *ABCB1* polymorphisms have been identified, with several SNPs shown to alter transporter expression, substrate specificity, and pharmacokinetics of drugs [52]. Among them, the synonymous SNP *ABCB1 3435C>T (rs1045642)* at exon 26 has been extensively studied [53, 54]. Although this variant does not alter the amino acid sequence, it has been linked to modifications in mRNA stability, protein conformation, and *P-gp* expression levels [55, 56]. Clinical studies suggest that the 3435C>T polymorphism modulates the risk and progression of several conditions, including epilepsy, Parkinson’s disease, and AD [57]. In the context of AD, reduced transporter efficiency linked to this variant could impair *Aβ* clearance [58], thereby facilitating its deposition and contributing to disease progression [59].

Previous research investigating the *ABCB1 3435C>T* (rs1045642) polymorphism in AD has yielded mixed findings. Some studies report associations between the T allele and increased susceptibility to AD or greater disease severity [60], while others fail to confirm significant links. These discrepancies may reflect ethnic differences, small sample sizes, and methodological variability. Importantly, while genetic studies have explored the variant’s association with cognitive decline and AD risk, far fewer have examined its relationship with neuropsychological manifestations of AD. Given that neuropsychological symptoms constitute a core component of disease burden, understanding genetic influences on their occurrence and severity is critical. To date, the role of the *ABCB1 3435C>T* (rs1045642) variant in modulating cognitive impairment symptoms in AD patients remains largely unexplored, representing a significant gap in knowledge.

**Fig. 1:**
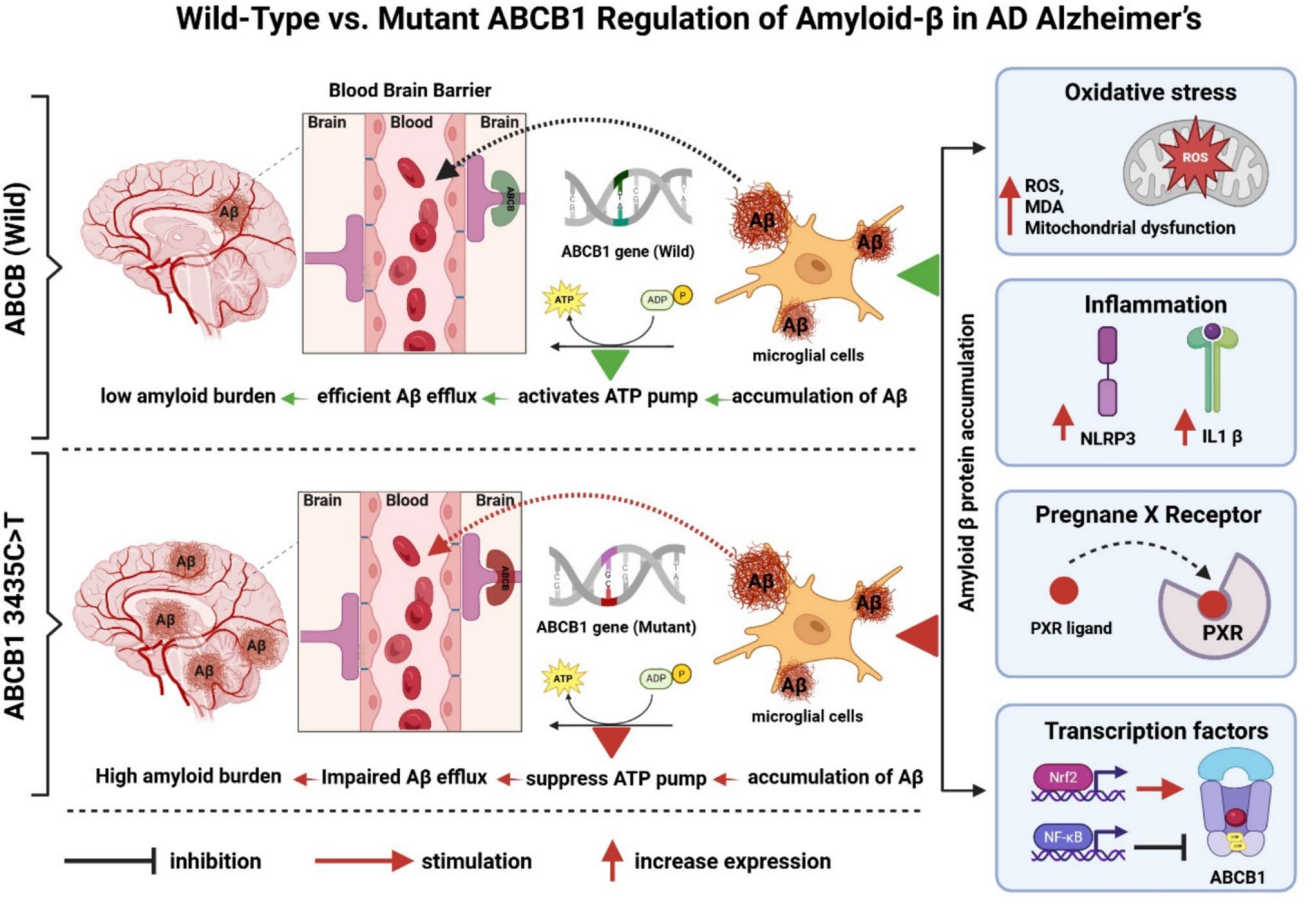
A schematic diagram illustrates the mechanistic pathway of *Aβ* accumulation and clearance in Alzheimer’s disease in relation to ABCB1 genotype. Oxidative stress (ROS, lipid peroxidation, mitochondrial dysfunction) and neuroinflammatory signaling (IL-1β, NLRP3 inflammasome activation) trigger intracellular cascades such as *NF-κB*, *MAPK/ERK*, *PI3K/Akt*, and *JNK*, which downregulate or impair *ABCB1* activity at the blood-brain barrier (BBB), thereby reducing Aβ efflux and promoting its accumulation within brain tissue. *NF-κB* activation through *RAGE–Aβ* interaction further amplifies inflammatory suppression of *ABCB1*, creating a feed-forward loop of Aβ deposition. In contrast, protective pathways, including nuclear receptor signaling (*PXR*) and antioxidant defense via *Nrf2*, upregulate *ABCB1* transcription and enhance efflux function, facilitating Aβ clearance. The impact of genetic variation is highlighted by the difference between wild-type *ABCB1* (3435C allele) and mutant-type (3435T allele). In wild-type *ABCB1* (3435C allele), transporter activity is preserved, supporting effective clearance, whereas the *ABCB1 3435C>T* (rs1045642) polymorphism impairs expression and function, leading to reduced efflux and greater amyloid accumulation in the brain. Together, these regulatory mechanisms integrate environmental stressors, transcriptional control, and genetic polymorphism to determine the balance between Aβ clearance and deposition in the Alzheimer’s brain. Abbreviations: *ABCB1* ATP-binding cassette subfamily B member 1, *ROS* reactive oxygen species, *IL1β* interleukin 1β, *NLRP3* NOD-LRR and pyrin domain-containing protein 3, *MDA* malondialdehyde, *NF-κB* nuclear factor kappa β, *PI3K* phosphatidylinositol-3-phosphate, *PXR* pregnane X receptor, *Nrf2* nuclear erythroid factor 2, *BBB* blood-brain barrier, *RAGE* receptor for advanced glycation end products, *ATP* adenosine-3-phosphate, *ADP* adenosine-3-phosphate, *P* phosphate

The present study aims to address this gap by investigating the association between the *ABCB1 3435C>T* (rs1045642) variant and both disease severity and neuropsychological symptoms in Egyptian patients with AD. Specifically, our objectives are (1) to assess the distribution of the *ABCB1 3435C>T* (rs1045642) polymorphism among Egyptian AD patients, (2) to evaluate its relationship with clinical severity as measured by standardized cognitive and functional scales, and (3) to examine its potential contribution to the emergence and severity of neuropsychological symptoms. Through clarifying these associations, this research aims to shed light on the genetic determinants contributing to Alzheimer’s disease (AD) variability within the Egyptian population, identify potential genetic markers for early detection of individuals at elevated risk, and support the advancement of personalized therapeutic approaches that address both the cognitive and psychological aspects of the disorder. The mechanistic pathway of *Aβ* accumulation and clearance in Alzheimer’s disease in relation to *ABCB1 3435C>T* (rs1045642) genotype is presented in Fig. 1.

## Methods

### Study Design and Ethical Considerations

This research employed a case–control observational genetic association design to investigate the correlation between the *ABCB1 3435C>T* (rs1045642) polymorphism and both the severity of AD and its related neuropsychological manifestations within an Egyptian population. Ethical approval was obtained from the Ethical Committee of the Faculty of Medicine, Ain Shams University (FMASU MS306/2024). All study procedures complied with the ethical standards outlined in the Declaration of Helsinki (2013 revision). Prior to participation, written informed consent was obtained from all subjects or their legally authorized representatives.

### Patient Recruitment and Sample Collection

Between May 2024 and May 2025, a total of 300 participants were recruited from the Geriatric and Neuropsychiatry Hospitals of Ain Shams University. The study population consisted of 150 patients clinically diagnosed with Alzheimer’s disease (AD) and 150 cognitively healthy controls, matched for age and sex. The diagnosis of AD was established according to the National Institute on Aging–Alzheimer’s Association (NIA-AA) criteria, based on comprehensive clinical evaluation, cognitive assessment, and neuroimaging when necessary. Control participants were thoroughly screened to exclude any evidence of dementia or neuropsychological illness [61]. From each subject, 3 mL of peripheral venous blood was collected in EDTA-coated tubes for DNA extraction, and the samples were promptly processed and stored at −20 °C until molecular analysis.

Eligible patients in the AD group were 60 years or older, had a confirmed diagnosis of probable Alzheimer’s disease, and were accompanied by a caregiver capable of providing reliable collateral information. Control participants were also aged 60 years or above, demonstrated normal cognitive performance, MMSE score > 26, and had no prior history of neurological or psychological disorders [62]. Participants in both groups were excluded if they had major psychological conditions (such as schizophrenia or bipolar disorder), neurological diseases other than AD (including Parkinson’s disease, stroke, or epilepsy), severe systemic illnesses (such as advanced hepatic, renal, or cardiac failure), alcohol or substance abuse, or were unable or unwilling to provide informed consent.

### Clinical and Neuropsychological Assessment

All participants diagnosed with Alzheimer’s disease underwent a comprehensive clinical and psychological evaluation using the MMSE and the Clinical Dementia Rating (CDR) scale. The MMSE is a 30-point cognitive screening tool designed to assess several domains, including orientation, registration, attention, calculation, recall, language, and visuospatial skills. Each correct answer is assigned one point, yielding a total score out of 30, where higher scores indicate superior cognitive performance. Conventionally, scores of 27–30 denote normal cognition, 21–26 suggest mild impairment, 10–20 reflect moderate impairment, and below 10 indicate severe cognitive decline [63].

The CDR scale, a clinician-rated structured instrument, evaluates six functional domains: memory, orientation, judgment and problem-solving, community affairs, home and hobbies, and personal care. Each domain is rated on a 5-point scale (0 = no dementia, 0.5 = questionable or very mild dementia, 1 = mild dementia, 2 = moderate dementia, 3 = severe dementia) [64]. A global CDR score is calculated using a standardized algorithm, with memory serving as the central reference domain. Higher scores correspond to greater dementia severity: CDR-0 represents normal cognition, CDR-0.5 denotes very mild impairment, CDR-1 indicates mild dementia, CDR-2 moderate dementia, and CDR-3 severe dementia [65]. Together, the MMSE and CDR provided a comprehensive and complementary evaluation of both quantitative cognitive performance and qualitative dementia severity in the study population [66].

### DNA Extraction

The QIAamp DNA Blood Mini Kit, cat. No. 51104, purchased from Qiagen, was used to isolate genomic DNA from whole blood samples using the QIAamp DNA Blood Mini Kit (Qiagen, Hilden, Germany; Cat. No. 51104) in accordance with the manufacturer’s standard protocol. Approximately 3 mL of EDTA-anticoagulated blood was used per extraction. The A260/A280 ratio > 1.8, measured spectrophotometrically by NanoDrop (Thermo Fisher Scientific, Waltham, MA, USA), confirms DNA purity and determines the actual concentration. Extracted DNA was stored at −20 °C for further testing [67].

### Genotyping of *ABCB1 3435C>T* (rs1045642)

The TaqMan allelic discrimination real-time PCR assay was utilized to determine the genotype for rs1045642 (Applied Biosystems, Thermo Fisher Scientific; Assay ID: C__7586657_20) together with TaqMan Genotyping Master Mix (Thermo Fisher Scientific; Cat. No. 4371355) in accordance with the manufacturer’s recommendations [68]. Amplification was performed on the StepOnePlus™ Real-Time PCR System (Applied Biosystems, Thermo Fisher Scientific). The PCR cycling conditions include 10 min for initial denaturation at 95 °C, followed by denaturation at 95 °C for 15 s and annealing/extension at 60 °C for 1 min for 40 cycles.

Allelic discrimination and genotype calling (CC, CT, and TT) were performed automatically using StepOne™ Software v2.3 (Applied Biosystems, Thermo Fisher Scientific).

### Statistical Analysis

The G*Power version 3.1.9.7 (Heinrich Heine University, Düsseldorf, Germany) was used to analyze the sample size. Based on a medium effect size (Cohen’s *w* = 0.3), an alpha level of 0.05, and a statistical power of 0.80, the minimum required sample size was calculated to be 88 participants per group. To compensate for potential dropouts or incomplete data, the sample size was increased to 150 AD patients and 150 controls, giving a total of 300 subjects.

Data analysis was performed by the *IBM SPSS Statistics for Windows, Version 25.0 (IBM Corp., Armonk, NY, USA)* and *GraphPad Prism Version 9.0 (GraphPad Software, San Diego, CA, USA)*. Continuous variables were summarized as mean ± standard deviation (SD) or median with interquartile range (IQR), depending on data distribution, which was evaluated using the Shapiro–Wilk test. Categorical variables, including genotype and allele frequencies, were expressed as counts and percentages, with group comparisons conducted via the chi-square (*χ*^2^) or Fisher’s exact test as appropriate. For between-group analyses, independent-samples *t*-tests or Mann–Whitney *U* tests were applied when comparing two groups, while one-way ANOVA or Kruskal–Wallis tests were used for comparisons across more than two groups, followed by suitable post hoc corrections.

Associations between *ABCB1 3435C>T* (rs1045642) genotypes and AD risk were assessed by odds ratios (ORs) with 95% confidence intervals (CIs) under different inheritance models (dominant, recessive, and additive). Logistic regression analyses were performed to adjust for confounding factors such as age and sex. The impact of genotypes on continuous clinical measures (MMSE and CDR scores) was evaluated by linear regression. Correlations were examined using Pearson’s or Spearman’s coefficients as appropriate. A two-tailed *p*-value < 0.05 was considered statistically significant. Graphical illustrations, including box plots, bar charts, and scatter plots, were generated with GraphPad Prism for data visualization.

## Results

### Baseline Demographic and Neuropsychological Profiles of Study Participants

The demographic and clinical data of all participants (*n* = 300) are presented in Table 1. The mean age of the AD cohort was 75.35 ± 6.91 years (range: 52–95), slightly lower but not significantly different from controls (77.78 ± 5.58 years, range: 65–95; *p* = 0.092). Gender distribution showed no statistical difference between groups, with males comprising 55.3% of the AD group and 44.7% of controls (*p* = 0.083). A family history of dementia was reported in 25.3% of patients. Psychological assessment of the AD cohort revealed median scores of 3.0 for memory loss, 2.0 for orientation, 2.0 for judgment, 1.0 for problem solving, and 1.0 for community affairs, while home hobbies demonstrated a median score of 1.0. The mean Clinical Dementia Rating (CDR) score was 2.0, classifying 31% of patients with mild dementia, 32% with moderate dementia, and 37% with severe dementia. The mean MMSE score among AD patients was 21.0, with 25.3% demonstrating preserved cognitive function (scores 27–30), 32.0% exhibiting mild impairment (21–26), 36.0% showing moderate impairment (10–20), and 6.7% classified as having severe cognitive impairment (scores below 10).

**Table 1:** Demographic and clinical characteristics of the studied cohorts

Variable	Control (*n* = 150)	AD (*n* = 150)	*p*-value
Age (years), mean ± SD, range	77.78 ± 5.58 (65–95)	75.35 ± 6.91 (52–95)	0.092
Gender: *n* (%)			
* Male* * Female*	68 (44.7) 82 (55.4)	84 (55.3) 66 (44.6)	0.083
Family history: *n* (%)			
* No* * Yes*		112 (74.7) 38 (25.3)	
Clinical assessment of dementia			
* Memory loss (median)* * Orientation (median)* * Judgment (median)* * Problem solving (median)* * Community affairs (median)* * Home hobbies (median)* * CDR (median)* * MMSE (median)*		3.00 2.00 2.00 1.00 1.00 1.00 2.0 21.0	
Clinical assessment of dementia			
* Mild dementia (CDR1* < *0.5–2)* * Moderate dementia (CDR1: 2≤3)* * Severe dementia (CDR3:* ≥ *3)*		47 (31) 48 (32) 55 (37)	
Mini-Mental State (MMSE) (***n***%)			
* Normal cognition (MMSE: 27–30)* * Mild cognitive impairment (MMSE: 21–26)* * Moderate cognitive impairment (MMSE: 10–20)* * Severe cognitive impairment (MMSE* < *10)*		38 (25.3) 48 (32.0) 54 (36.0) 10 (6.7)	

*AD* Alzheimer’s disease, *Control* cognitively intact, *CDR* Clinical Dementia Rating Scale (0–5), *MMSE* Mini-Mental State Examination (0–30)

### Differential Distribution of* ABCB1 3435C>T *(rs1045642) Genotypes in AD and Cognitively Intact Cohorts

**Fig. 2:**
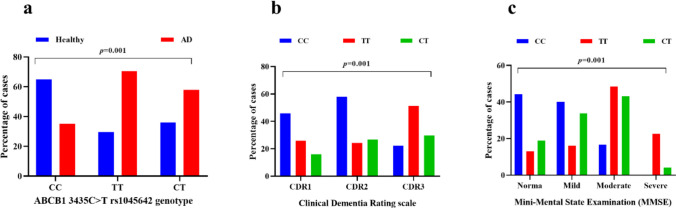
Comparative distribution of *ABCB1 3435C>T* (rs1045642) genotypes (CC, TT, CT) between healthy controls and AD patients (**a**); AD patients with different CDR scales (**b**); AD patients with different grades of impaired cognitive disorders as measured by MMSE (**c**). Data are expressed as the percentage distribution of cases within each group. Statistical comparison was performed using the chi-square test: *CDR* Clinical Dementia Rating “CDR1 (0.5–2): mild dementia, CDR2 (> 2–< 3): moderate dementia, CDR3 (≥ 3): severe dementia”; *MMSE* Mini-Mental State Examination “MMSE (27–30): normal cognition, MMSE (21–26): mild cognitive impairment, MMSE (10–20): moderate cognitive impairment, MMSE (< 10): severe cognitive impairment”

Table 2 and Fig. 2a display the genotypic distribution of the *ABCB1 3435C>T* (rs1045642) polymorphism among patients with Alzheimer’s disease (AD) and cognitively intact controls. A significant difference in genotype frequencies was observed between groups (*p* = 0.001). The homozygous wild-type CC genotype was more prevalent in the control cohort (64.8%) compared with AD patients (35.2%). Conversely, the homozygous TT genotype was markedly enriched in the AD group (70.5%) relative to controls (29.5%). The heterozygous CT genotype demonstrated an intermediate distribution, occurring in 57.8% of AD cases and 36.0% of controls. These findings indicate a strong association between the TT genotype and increased susceptibility to Alzheimer’s disease, while the CC genotype appeared more protective, being more frequent in healthy controls.

**Table 2:** Genotypic distribution of *ABCB1 3435C>T* (rs1045642) variant among all participants

Genotype variant	Control (*n* = 150)	AD (*n* = 150)	*p*-value
Homozygous CC: 128 (42.7)^a^	83 (64.8)^b^	45 (35.2)^b^	0.001
Homozygous TT: 44 (14.7)^a^	13 (29.5)^b^	31 (70.5)^b^	
Heterozygous CT: 128 (42.7)^a^	54 (36.0)^b^	74 (57.8)^b^	

Comparative analysis was conducted using the chi-square test

^a^Percentage is calculated within the *ABCB1 3435C>T (rs1045642)* genotype “Homozygous CC, Homozygous TT, Heterozygous CT”

^b^Percentage is calculated within the same group “Healthy, cognitively intact

### Impact of* ABCB1 3435C*>*T *(rs1045642) Polymorphism on Dementia Progression in Alzheimer’s Disease

Table 3 and Fig. 2b examine the relationship between the *ABCB1 3435C>T* (rs1045642) polymorphism and dementia severity in patients with Alzheimer’s disease, stratified by Clinical Dementia Rating (CDR) scores. A significant association was identified between genotype distribution and dementia stage (*p* = 0.005). Among patients with mild dementia (CDR1: 0.5–2) and moderate dementia (CDR2: > 2, < 3), the homozygous CC genotype was most frequent (45.9% and 58.1%, respectively), while TT and CT were less represented in both groups. For severe dementia (CDR3: ≥ 3), the TT genotype continued to show the highest prevalence (51.1%), compared with 22.2% for CC and 29.7% for CT. Collectively, these results suggest that carriage of the TT genotype is linked to more advanced stages of dementia, whereas the CT genotype is more commonly observed in earlier disease stages, and the CC genotype appears underrepresented in severe dementia cases.

**Table 3:** Genotypic distribution of *ABCB1 3435C>T* (rs1045642) variants among AD patients stratified by the severity of Clinical Dementia Rating scale (CDR)

Genotype variant	Homozygous CC (*n* = 45, 30.0%)	Homozygous TT (*n* = 31, 20.7%)	Heterozygous CT (*n* = 74, 49.3%)	*p*-value
CDR1 (0.5–2): 47 (31)^a^	34 (45.9)^b^	8 (25.8)^b^	5 (16.1)^b^	0.005
CDR2 (> 2–3): 48 (32)^a^	18 (58.1)^b^	18 (24.3)^b^	12 (26.7)^b^	
CDR3 (> 3): 55 (37)^a^	10 (22.2)^b^	23 (51.1)^b^	22 (29.7)^b^	

Comparative analysis was conducted using the chi-square test

^a^Percentage is calculated within the *ABCB1 3435C>T* (rs1045642) genotype “Homozygous CC, Homozygous TT, Heterozygous CT”

^b^Percentage is calculated within the same group: “CDR1 (0.5–2): mild dementia, CDR2 (> 2–< 3): moderate dementia, CDR3 (≥ 3): severe dementia”

### Impact of *ABCB1 3435C*>*T *(rs1045642) Variant on Cognitive Decline Stratified by MMSE

Table 4 and Fig. 2c illustrate the distribution of *ABCB1 3435C>T* (rs1045642) genotypes among Alzheimer’s disease patients stratified by MMSE categories. A highly significant association was observed between genotype frequencies and cognitive status (*p* = 0.001). Among patients with normal cognition (MMSE: 27–30), the CC genotype was most frequent (44.4%), followed by CT (18.9%) and TT (12.9%). In those with mild cognitive impairment (MMSE: 21–26), CC carriers again predominated (40.0%), with CT (33.8%) and CC (16.0%) genotypes occurring less often. A shift in distribution was evident in the moderate impairment group (MMSE: 10–20), where the TT genotype became most common (48.4%), followed by CT (43.2%) and CC (15.6%). In patients with severe impairment (MMSE < 10), no CC genotypes were detected, while TT (22.6%) and CT (4.1%) genotypes remained present. These findings suggest that the CC genotype is linked to preserved or mildly impaired cognition, whereas the CT genotype becomes more frequent in moderate impairment, and the presence of TT is characteristic of severe cognitive decline. This distribution highlights a potential role of the *ABCB1 3435C>T* (rs1045642) variant in modulating the severity of cognitive impairment in Alzheimer’s disease.

**Table 4:** Genotypic distribution of *ABCB1 3435C>T* (rs1045642) variants among AD patients stratified by the severity of MMSE

Genotype variant	Homozygous CC (*n* = 45, 30.0%)	Homozygous TT (*n* = 31, 20.7%)	Heterozygous CT (*n* = 74, 49.3%)	*p*-value
MMSE (27–30): 38 (25.3)^a^	20 (44.4)^b^	4 (12.9)^b^	14 (18.9)^b^	0.001
MMSE (21–26): 48 (32.0)^a^	18 (40.0)^b^	5 (16.0)^b^	25 (33.8)^b^	
MMSE (10–20): 54 (36.0)^a^	7 (15.6)^b^	15 (48.4)^b^	32 (43.2)^b^	
MMSE (< 10): 10 (6.7)^a^	0 (0)	7 (22.6)^b^	3 (4.1)^b^	

The chi-square test was utilized for the comparative analysis between groups

^a^Percentage is calculated within the *ABCB1 3435C>T* (rs1045642) genotype “Homozygous CC, Homozygous TT, Heterozygous CT”

^b^Percentage is calculated within the same group: “MMSE (27–30): normal cognition, MMSE(21–26): mild cognitive impairment, MMSE (10–20): moderate cognitive impairment, MMSE(< 10): severe cognitive impairment”

### Association of* ABCB1 3435C*>*T *(rs1045642) Genotypes with Alzheimer’s Disease Risk and Severity: Logistic Regression and Odds Ratio Analysis

**Fig. 3:**
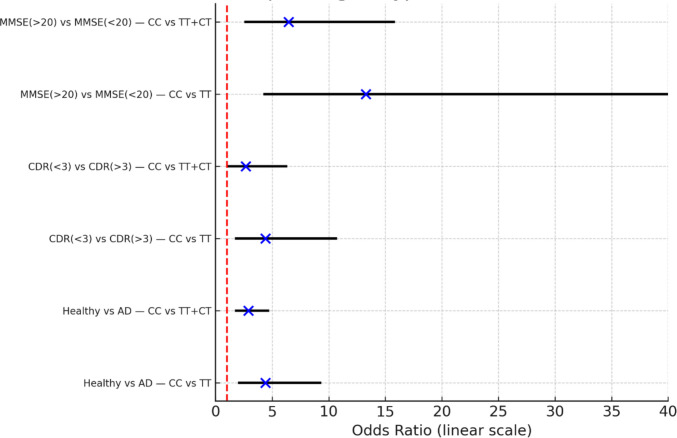
Forest plot of odds ratios (OR) with 95% confidence intervals (CI) for associations between the *ABCB1 3435C>T* (rs1045642) genotype and clinical outcomes. Logistic regression analysis was performed using CC genotypes as reference categories. The CC genotype was significantly associated with a higher likelihood of being in the healthy control group versus Alzheimer’s disease (AD), having a lower Clinical Dementia Rating (CDR < 3), and achieving higher MMSE > 20 scores. ORs are plotted on a linear scale (0–40), with the dashed vertical line indicating the null value (OR = 1).

In Table 5 and Fig. 3, logistic regression analysis revealed a significant and consistent association between the CC genotype of the *ABCB1 3435C>T* (rs1045642) polymorphism and more favorable clinical outcomes. When comparing healthy controls to AD patients, individuals carrying the CC genotype showed markedly increased odds of being in the healthy group, whether contrasted against TT alone (OR = 4.39, 95% CI 2.02–9.51, *p* < 0.001) or the combined TT + CT group (OR = 2.71, 95% CI 1.73–4.23, *p* < 0.001). Similarly, within AD patients, CC carriers were significantly more likely to fall into the lower CDR category (< 3), reflecting milder disease severity, compared to TT (OR = 4.40, 95% CI 1.73–11.20, *p* = 0.002) or TT + CT (OR = 2.66, 95% CI 1.19–5.92, *p* = 0.017). The strongest associations were observed in relation to cognitive performance and disease severity, where CC carriers exhibited substantially higher odds of having an MMSE score > 20, again relative to TT (OR = 13.27, 95% CI 4.66–37.80, *p* < 0.001) and TT + CT (OR = 6.45, 95% CI 2.71–15.34, *p* < 0.001). Taken together, these findings strongly support a protective role of the CC genotype, associating it with better preserved cognitive performance and less severe dementia manifestations as assessed by MMSE and CDR scores.

**Table 5:** Association of *ABCB1 3435C>T* (rs1045642) genotypes with Alzheimer’s disease outcomes, presented as odds ratios (OR) and 95% confidence intervals (CI)

Comparison	Group 1	Group 2	OR	95% CI	*p*-value
Healthy vs AD CC vs TT	CC: 83 (64.8%) TT: 13 (29.5%)	CC: 45 (35.2%) TT: 31 (70.5%)	4.398	2.09–9.24	0.0001
Healthy vs AD CC vs TT + CT	CC: 83 (64.8%) TT + CT: 67 (39%)	CC: 45 (35.2%) TT + CT: 105 (61%)	2.89	1.79–4.64	0.0001
CDR (< 3) vs CDR (≥ 3) CC vs TT	CC: 52 (55%) TT: 26 (27%)	CC: 10 (18%) TT: 22 (40%)	4.4	1.82–10.64	0.0001
CDR(< 3) vs CDR (> 3) CC vs TT + CT	CC: 52 (55%) TT + CT: 43 (45%)	CC: 10 (18%) TT + CT: 22 (40%)	2.66	1.14–6.22	0.025
MMSE (> 20) vs MMSE (< 20) CC vs TT	CC: 38 (84%) TT: 9 (29%)	CC: 7 (16%) TT: 22 (71%)	13.2	4.33–40.62	0.0001
MMSE (> 20) vs MMSE (< 20) CC vs TT + CT	CC: 38 (84%) TT + CT: 48 (46%)	CC: 7 (16%) TT + CT: 57 (54%)	6.45	2.64–15.74	0.0001

The logistic regression analysis, complemented by Fisher’s exact test, revealed a significant association between the *ABCB1 3435C>T* (rs1045642) genotype and Alzheimer’s disease outcomes. Across all comparisons, the CC genotype is associated with higher odds of being in the “more favorable” clinical group.

- Healthy (vs AD): TT carriers had a 4.40-fold higher risk of AD compared to CC (*p* < 0.001), while TT + CT combined also showed increased odds (OR = 2.89, *p* < 0.001).
- Lower CDR (milder dementia) vs higher CDR (severe): CC associates with lower CDR categories; effects are significant and clinically meaningful (ORs: 2.7–4.4).
- Higher MMSE (> 20) vs lower MMSE (< 20): The strongest associations were observed; TT carriers had a 13.27-fold higher likelihood of scoring < 20 compared to CC (*p* < 0.001), while TT + CT carriers had a 6.45-fold higher risk (*p* < 0.001). The strongest associations are observed here; CC shows large effect sizes (OR: 6.5–13.3), consistent with better cognitive performance.

Taken together, these findings demonstrate that the TT genotype is a major risk factor for AD susceptibility and cognitive decline, evident in CDR severity progression and MMSE-based impairment**.**

Odds ratios (ORs) and 95% confidence intervals (CIs) were calculated using Fisher’s exact test. Group 1 represents the reference clinical or cognitive category “healthy controls, milder dementia (CDR < 3), or better cognitive performance (MMSE > 20), while Group 2 represents the comparison group “Alzheimer’s disease patients, more severe dementia (CDR ≥ 3), or poorer cognitive performance (MMSE < 20).” An OR > 1 indicates increased odds in Group 1 relative to Group 2. Dementia severity and cognitive performance were classified using standard CDR and MMSE cutoffs

## Discussion

Alzheimer’s disease (AD) is a progressive neurodegenerative condition driven by a multifaceted interaction between genetic predisposition and environmental influences [69, 70]. While much is known about common genetic risk factors such as APOE, less attention has been paid to genes involved in blood-brain barrier function and *Aβ* clearance [71], particularly in non-European populations [72]. The *ABCB1* gene, encoding the *P-gp* efflux transporter [73], is of particular interest given its role in limiting neurotoxic accumulation in the brain [70]. Variants in the *ABCB1 3435C>T* (rs1045642) polymorphism have been reported to alter transporter expression and functionality, but available evidence has been inconsistent and limited in scope, especially in Middle Eastern cohorts [74]. To address this gap, we investigated the association of this polymorphism with AD susceptibility and disease severity in an Egyptian population of 300 participants, including both healthy controls and patients with clinically diagnosed AD. Genotyping was conducted using PCR, and associations were evaluated in relation to both Clinical Dementia Rating (CDR) scores and MMSE performance.

The results of this study revealed striking differences in genotype distribution between healthy controls and AD patients. The CC genotype was notably frequent among cognitively intact subjects, while the TT genotype predominated in the AD group. The heterozygous CT genotype displayed an intermediate distribution, suggesting a potential dose effect of the T allele. Within AD patients, genotypic patterns correlated strongly with disease severity. The CC genotype was enriched in those with mild or moderate dementia, while TT carriers were disproportionately represented in severe dementia cases. Similarly, analysis of MMSE categories demonstrated that CC carriers were more frequently observed among those with preserved or mildly impaired cognition, whereas TT carriers dominated in groups with moderate or severe impairment. Logistic regression analysis confirmed these associations, demonstrating that TT carriers had over four times the risk of developing AD compared to CC carriers and more than a 13-fold increased risk of scoring below 20 on the MMSE, consistent with advanced cognitive decline. Together, these findings indicate that the TT genotype confers substantial risk for disease susceptibility and progression, while the CC genotype may be protective.

When compared with prior literature, our findings are broadly consistent with studies reporting that the TT genotype is associated with decreased *P-gp* expression and reduced *Aβ* clearance, thereby predisposing to greater cognitive impairment [75, 76]. The association between T allele carriage and increased AD risk was previously reported in several European and Asian studies, supporting our observation of TT as a high-risk genotype. Ben Halla et al. reported that the CT and TT genotypes of the *ABCB1 3435C>T* (rs1045642) polymorphism were more frequent among healthy controls than in AD patients, a finding that is not consistent with our results [77]. In contrast, an original European case–control study reported an increased risk of AD among CC genotype carriers, with no protective effect observed for the T allele [75]. Collectively, these conflicting findings highlight substantial heterogeneity across populations. Such discrepancies may be explained by differences in genetic background, allele frequencies, and linkage disequilibrium patterns, as well as variations in sample size, diagnostic criteria, clinical characterization, and cognitive assessment tools. Our findings add to this body of evidence by demonstrating a clear and clinically relevant association in an Egyptian cohort, emphasizing the importance of population-specific genetic studies in elucidating AD risk.

An important aspect of the present study lies in its novelty. While most prior research has primarily assessed disease susceptibility, we extended our analysis to explore the impact of the *ABCB1 3435C>T* (rs1045642) variant on disease severity and cognitive impairment using both CDR staging and MMSE stratification. This dual approach allowed us to capture a more nuanced relationship between genotype and clinical phenotype. We observed a stepwise distribution, with CC carriers more likely to remain cognitively preserved, CT carriers clustering in intermediate stages of impairment, and TT carriers driving progression to severe disease. Such a clear genotype–phenotype correlation has rarely been described in Middle Eastern populations and underscores the potential role of *ABCB1* genotyping as a clinical tool in risk stratification.

Despite its strengths, this study has several limitations. It was conducted at a single center with a moderate sample size, and larger multicenter cohorts are required to confirm the robustness of the findings. Although diagnosis was established using recognized clinical criteria, no cerebrospinal fluid biomarkers or amyloid PET imaging were performed; therefore, a biological definition of Alzheimer’s disease according to the 2018 NIA-AA AT(N) framework was not available, potentially introducing diagnostic heterogeneity. While CDR and MMSE are widely utilized instruments, they may not comprehensively capture neuropsychiatric manifestations and can be influenced by educational and cultural variables. The genetic analysis was limited to the *ABCB1 3435C>T variant* (rs1045642), and additional polymorphisms within *ABCB1* or genes involved in amyloid processing, vascular regulation, or blood-brain barrier integrity were not assessed. Moreover, environmental and pharmacological factors affecting *P-gp* activity were not systematically controlled. Finally, functional assays were not performed to directly evaluate transporter activity, limiting mechanistic interpretation of the observed genotype–phenotype associations.

To sum up, the study provides compelling evidence that the *ABCB1 3435C>T* (rs1045642) polymorphism influences both the risk and severity of Alzheimer’s disease in Egyptian patients. The TT genotype emerged as a strong risk factor, being consistently associated with advanced dementia stages and severe cognitive impairment, while the CC genotype appeared protective and was enriched among cognitively intact or mildly impaired individuals. These results emphasize the clinical relevance of *ABCB1 3435C>T* (rs1045642) genotyping as a potential biomarker for identifying individuals at greater risk of disease progression. Future studies involving larger and more diverse cohorts, as well as functional analyses, are required to further elucidate the underlying mechanistic pathways. Ultimately, this line of research may contribute to the development of more personalized approaches in the prediction, diagnosis, and management of AD.

## Conclusions

This study demonstrates that the *ABCB1 3435C>T* (rs1045642) polymorphism significantly influences both susceptibility to AD and the association with cognitive decline in Egyptian patients. The increased risk for AD, more advanced dementia stages, and severe cognitive impairment were strongly associated with the dominant homozygous TT genotype, whereas the CC genotype conferred relative protection, being more frequent among healthy and mildly impaired individuals. These results highlight the promise of *ABCB1 3435C>T* (rs1045642) genotyping as a potential genetic biomarker for both risk assessment and disease monitoring in AD. To confirm these associations and evaluate their clinical utility in the context of personalized AD management, further large-scale, multicenter studies integrated with functional analyses are essential.

## Data Availability

The original data is available upon request from the corresponding author.

## Abbreviations

AD: Alzheimer’s disease
PCR: Polymerase chain reaction
CDR: Clinical dementia rating
MMSE: Mini-mental state examination
OR: Odds ratios
CI: Confidence intervals
SNP: Single-nucleotide polymorphisms
BBB: Blood-brain barrier
BPSD: Behavioral and psychological symptoms of dementia
NMDA: N-Methyl-D-aspartate
Aβ: Amyloid-β
SD: Standard deviation
IQR: Interquartile range
